# Decision-Making at the Limit of Viability in Newborns: Insights from a Survey of Medical Professionals

**DOI:** 10.3390/children13081111

**Published:** 2026-08-19

**Authors:** Claudiu Voic, Melinda Ildiko Mitranovici, Zoran Laurentiu Popa, Maria Cezara Muresan, Septimiu Voidazan, Elena Silvia Bernad

**Affiliations:** 1 Doctoral School, Victor Babes University of Medicine and Pharmacy, 300041 Timisoara, Romania; claudiu.voic@umft.ro; 2 Department of Obstetrics and Gynecology, Emergency County Hospital Hunedoara, 331057 Hunedoara, Romania; 3 Doctoral School of Medicine and Pharmacy, George Emil Palade University of Medicine, Pharmacy, Science, and Technology of Targu Mures, 540142 Targu Mures, Romania; 4 Department of Obstetrics and Gynecology, Faculty of Medicine, Victor Babes University of Medicine and Pharmacy, 300041 Timisoara, Romania; popa.zoran@umft.ro (Z.L.P.); muresan.maria@umft.ro (M.C.M.); bernad.elena@umft.ro (E.S.B.); 5 Clinic of Obstetrics and Gynecology, Laparoscopy, In Vitro Fertilization and Embryotransfer Research Center, Pius Brinzeu County Clinical Emergency Hospital, 300723 Timisoara, Romania; 6 Center for Laparoscopy, Laparoscopic Surgery and In Vitro Fertilization, Faculty of Medicine, Victor Babes University of Medicine and Pharmacy, 300041 Timisoara, Romania; 7 Department of Epidemiology, “George Emil Palade” University of Medicine, Pharmacy, Sciences and Technology, 540142 Targu Mures, Romania; septimiu.voidazan@umfst.ro; 8 Center for Neuropsychology and Behavioral Medicine, Victor Babes University of Medicine and Pharmacy, 300041 Timisoara, Romania

**Keywords:** limit of viability, prematurity, survey, ethical issues, pregnancy at risk outcomes

## Abstract

**Highlights:**

**What are the main findings?**

- Variability in clinician perceptions on this topic.
- Value of continuing medical education.

**What are the implications of the main findings?**

- Shifts in perception and management after completing the course.
- Multidisciplinary approach, ethical considerations, and value of parental wishes in decision-making.

**Abstract:**

Legal definitions of human viability vary worldwide. Background/Objectives: The treatment of periviable newborns remains controversial and raises ethical concerns. However, currently, the World Health Organization (WHO) sets the lower limit of viability at 22 weeks of gestation or 500 g birth weight. The aim of our research was to determine the knowledge of healthcare practitioners in our country regarding this topic, as well as including a course after which we sought to evaluate whether their perceptions regarding the management of these cases changed. Methods: The recruitment of participants encompassed those who voluntarily took part in the survey, and 30 clinicians were included; they participated in a 30 h online course and completed a survey questionnaire before and after the course. Results: After Holm adjustment, we observed an improvement in participants’ perceptions regarding recommendations concerning a multidisciplinary approach, perinatal counseling (20.0% vs. 100.0%), ethical issues (16.7% vs. 93.3%), and awareness of national guidelines (36.7% vs. 100.0%). Moreover, they recognized the importance of including parental wishes in decision-making (Holm-adjusted *p* = 0.0469), but with clinicians having the final word in the decision. Other factors did not change, with practitioners having knowledge about the serious outcomes of newborns in such challenging situations. Conclusions: Ethically grounded strategies to guide the management of newborns at the limit of viability is of great importance, whereby gestational age is an insufficient parameter. A collaborative, multidisciplinary process is mandatory in order to ensure the best outcomes for the newborn. The value of continuing medical education should be acknowledged.

## 1. Introduction

Legal definitions of human viability vary in different countries, remaining a major challenge. Providers are often in a difficult position when faced with the delivery of an extremely premature infant [1]. This ethical issue places the neonatologist in a complicated position regarding whether to attempt resuscitation when faced with the delivery of an infant classically perceived to be in the “gray zone” of human viability [1,2]. The treatment of periviable newborns remains controversial and raises ethical concerns [3]. Additionally, there are variations regarding the gestational age threshold between physicians, and survey questionnaires have been developed to identify their views on this topic [4]. The definition of the limit of viability is not clear, with gestational age not being an independent factor and with the likelihood of survival outside the uterus having great importance [3]. Nevertheless, currently, the World Health Organization (WHO) sets the lower limit of viability at 22 weeks of gestation, a birth weight of 500 g, or a birth length of 25 cm [5]. The threshold for viability should be considered based on a combination of gestational age, fetal weight, genetics, and the availability of neonatal intensive care facilities. Despite the WHO’s threshold of 22 weeks of gestation, a fetus should be considered viable typically at around 23–24 weeks with appropriate medical intervention [6,7,8]. There is a need for greater uniformity and clarity in these aspects to avoid misinterpretation, as there is an international consensus that the threshold of viability is 23^+0^ weeks to 24^+6^ weeks of gestation. This raises major medical and ethical challenges [6,8]. The confusion surrounding the viability threshold reflects challenges in distinguishing between preterm birth and abortion. The classification of abortion and extreme preterm birth needs standardization [6]. In our country, the limit is set at 24 weeks; the guidelines do not consider ethical issues or perinatal counselling with a multidisciplinary approach in the management of pregnant women [3]. It is crucial to separate the definition/reporting threshold from the clinical threshold for active treatment/resuscitation.

There are situations in which the interests of the parents and the infant may not coincide with those of the healthcare professionals, which makes the decision even more challenging [1]. Physicians’ decisions can be influenced not only by their personal knowledge of the laws but also by their own moral values and religious beliefs [1].

At present, the reported threshold for resuscitation is lower, with active treatment for infants starting from <23 weeks of gestation [2]. However, periviable infants represent a great burden for healthcare systems worldwide. Early gestation is associated with worse outcomes; however, outcomes vary between and within countries, as well as between different levels of hospitals [2].

While the American College of Obstetricians and Gynecologists (ACOG) indicates that physicians should never violate a woman’s basic right to personal autonomy [7,9], it should be noted that, in this case, the patient is the neonate, who is unable to exercise autonomy. This means that such complex decisions require a dialog between the healthcare practitioner and the parents to ensure the best outcome for the infant. The ACOG and the American Academy of Pediatrics (AAP) guidelines recommend that the management of pregnant women be based on shared decision-making and informed consent [7,9,10], but this recommendation is not consistently applied [10].

Many guidelines give only suggestions, being vague and ambiguously worded, while many countries have no periviability guidelines [10].

The aim of our study was to determine the knowledge of healthcare practitioners in our country regarding the limit of viability threshold in newborns, as well as to implement a course after which we sought to evaluate whether their perceptions regarding the management of these cases changed.

## 2. Materials and Methods

The recruitment of participants encompassed those who voluntarily took part in the survey. However, only a limited number of healthcare professionals agreed to complete the questionnaire. An electronic survey was sent to 50 obstetricians and neonatologists from several hospitals. The survey questions included the demographic data of the respondents: the current location of the practice, years in practice, religious affiliations, and knowledge of local legal statutes and definitions. The inclusion criteria included participating in an online course, followed by completing the same questionnaire to assess whether they had changed their approach. The exclusion criteria consisted of loss to follow-up or refusal to participate in the course. This study was conducted on a small sample size in the form of a pre-/post-course study. Out of the 50 people invited, 38 completed the first questionnaire, while only 30 completed the course and the second questionnaire. Ultimately, we included 30 participants, who answered 17 questions. We blinded the participants so that they did not know the goals of the study. An informed consent form was signed by all participants. They completed a 30 h online course followed by the same survey. Data collection was conducted between June 2026 and July 2026.

The main study question concerned the lowest gestational age at which providers would recommend resuscitation and treatment, whether they considered parents’ wishes in the final decision, and whether they would agree to a multidisciplinary approach in perinatal counselling. Other questions explored respondents’ conceptual understanding of viability. No single, universally accepted “standardized questionnaire” exists for the limit of viability because clinical guidelines vary significantly by region and evolve as medical technology improves [2].

The course model was based on the questionnaire and was divided into the following workshops (Table 1).

The entire survey is available in the Supplementary Materials. Approval was obtained from the Institutional Ethics Committee of the County Hospital Alexandru Simionescu Hunedoara before the implementation of this study (Number 8452/8 June 2026).

Responses were exported to an Excel database (Microsoft, San Francisco, CA, USA).

### Statistical Analyses

Statistical analyses were performed using the R statistical software version 4.6.1 (R Foundation for Statistical Computing, Vienna, Austria). Categorical variables were summarized as absolute frequencies and percentages. Because the same participants completed the questionnaire before and after the educational intervention, pre- and post-course observations were analyzed as paired data. Binary paired outcomes were assessed using the exact McNemar test, while multi-categorical paired outcomes were evaluated using the Stuart–Maxwell test of marginal homogeneity. Individual pre–post transitions and absolute changes in proportions with 95% confidence intervals were reported to describe the magnitude and precision of the observed changes. To account for multiple testing, *p* values were adjusted using the Holm procedure. Given the small sample size and sparse demographic subgroups, participant characteristics were summarized descriptively without inferential subgroup comparisons. All tests were two-sided, and a Holm-adjusted *p* value < 0.05 was considered statistically significant.

While 38 individuals completed the first questionnaire, only 30 participants completed the course; this situation led to dropout bias. This could reduce the study’s statistical power and limits how well the findings apply to all doctors. After checking the first set of questionnaire answers, we observed that the doctors who left the study shared the same characteristics, i.e., age, experience, or initial knowledge, as those who remained. Moreover, we noted the number of doctors who left.

## 3. Results

Participants’ demographic and professional characteristics are summarized descriptively in Table 2. Given the small sample size and sparse data within several demographic and professional categories, no inferential subgroup comparisons were performed.

Significant within-participant changes were observed for all three gestational-age-related items. Following the course, all participants selected 24 weeks as the limit of viability threshold (Q1), compared with 46.7% before training. Knowledge of the WHO’s lower viability threshold also increased, with all participants selecting 22 weeks after training, compared with 63.3% before training. These changes remained statistically significant after Holm correction for multiple comparisons (Table 3).

Significant changes were observed in several binary questionnaire items after the course (Table 4). Parental involvement in treatment decisions increased from 60.0% to 90.0% (absolute change: +30.0 percentage points), with 10 participants’ responses changing from “no” to “yes” and one from “yes” to “no” (Holm-adjusted *p* = 0.0469). The largest changes were observed for perinatal counselling (20.0% vs. 100.0%), recognition of the importance of ethical considerations (16.7% vs. 93.3%), and awareness of national guidelines (36.7% vs. 100.0%); all remained statistically significant after Holm correction (adjusted *p* < 0.0001). The importance of economic costs did not remain significant after adjustment (*p* = 0.0625), while the change regarding neonatologist stress was not significant (*p* = 0.1250). The proportion considering the infant’s appearance in the delivery room an appropriate prognostic tool decreased from 33.3% to 6.7% and remained significant after adjustment (*p* = 0.0391).

Significant changes in the distribution of responses were also observed for the multi-categorical items (Table 5). For Q10, 83.3% of participants changed their response category, with 96.7% selecting both pain and long-term distress among infants in their decision-making after the course, compared with 16.7% before the course (Holm-adjusted *p* < 0.0001). For Q13, 66.7% changed their response, and the proportion that selected training for obstetricians, neonatologists, and midwives as mandatory for future approaches increased from 23.3% to 90.0% (adjusted *p* = 0.00045). Significant redistribution was also observed for Q14 (adjusted *p* = 0.00186), indicating that many environmental factors influenced the clinicians’ decisions. Although the responses to Q12 changed after the course, the difference did not remain statistically significant after correction for multiple comparisons (adjusted *p* = 0.0549).

For Q5 (*What are the main morbidities in periviable infants?*), pre-course responses were heterogeneous, with participants identifying different individual or combined complications associated with extreme prematurity. After the intervention, all participants (30/30, 100%) selected the comprehensive response encompassing respiratory, neurological, gastrointestinal, cardiovascular, and visual complications (Table 6).

For Q6 (*When would you suggest withdrawal of life-sustaining therapies?*), pre-course responses were distributed across several combinations of clinical and parental factors. After the course, 27 participants (90.0%) selected the combined response including a condition incompatible with life, severe brain injury, severe lung disease, and parental request. Because the response categories recorded before and after the course were not directly equivalent, these two items were interpreted descriptively and were not subjected to paired inferential testing (Table 7).

## 4. Discussion

Several survey questionnaires have been developed for healthcare professionals in order to analyze their views on the newborn limit of viability threshold and the appropriate management approach in this situation [3]. In our study, several data were recorded regarding participants’ features, including their demographic characteristics, religious views, and professional experience. Based on the questionnaire used to identify their opinions related to this topic, before and after the training session, we observed a statistically significant improvement in their knowledge regarding the international threshold imposed by the WHO, i.e., the gestational age at which treatment should be initiated (*p* < 0.0001) (Table 3).

After Holm adjustment, we observed an improvement in the participants’ perceptions regarding recommendations concerning a multidisciplinary approach, perinatal counseling (20.0% vs. 100.0%), ethical issues (16.7% vs. 93.3%), and awareness of national guidelines (36.7% vs. 100.0%). Moreover, they recognized the importance of including parental wishes in decision-making (Holm-adjusted *p* = 0.0469), but with clinicians having the final word in the decision (adjusted *p* = 0.0549), but these did not significantly change. The rating of the infant’s appearance in the delivery room as an appropriate prognostic tool decreased significantly (*p* < 0.0391). Significant changes in the distribution of responses were also observed (Table 5), such as considering both pain and long-term distress in the infant in decision-making (Holm-adjusted *p* < 0.0001); medical education for obstetricians, neonatologists, and midwives as a future approach (adjusted *p* = 0.00045); and environmental factors having an influence on the decision (adjusted *p* = 0.00186). Responses regarding many other factors did not exhibit changes, such as economic costs or neonatologist stress. Practitioners demonstrated knowledge about the serious outcomes of newborns in such challenging situations; after the course, they selected the comprehensive response. For Q6 (*When would you suggest withdrawal of life-sustaining therapies?*), after the course, 90.0% of participants selected a combination of comorbidities incompatible with life, including also parental requests, which was interpreted descriptively. We observed an immediate post-course change in the questionnaire responses, but, in the future, careful monitoring of changes in the attitudes of healthcare professionals regarding therapeutic decisions for infants at the limit of viability is required. Moreover, gestational age is not an independent factor influencing the limit of viability threshold.

The gray zone of gestational age has remained unchanged over the past several years, whereby the age at which perinatal care should be offered ranges from 22 to 25 weeks. It remains an ongoing debate among perinatal physicians. Attitudes vary among practitioners in different countries and among individuals in the same country [11]. Several factors are involved in management decisions; in addition, most parents ignore the complexity of care during and after hospitalization. It is crucial to inform parents about the survival rates and the risks of long-term disability. Data in this area are still insufficient [11]. It is also worth noting that long-term neurodevelopmental, cognitive, and sensory outcomes should also be incorporated into counselling concerning infants born at the threshold of viability [12]. Such decisions can be particularly challenging in developing countries with suboptimal resources [13].

We adapted questionnaires from different surveys. This framework enables the comparison of interdisciplinary gaps and the measurement of differences in attitudes between obstetricians and neonatologists. Its key feature is that it focuses heavily on the “gray zone” in the decision-making process, navigating between personalized approaches and standardization [14]. Questionnaires should include dedicated sections quantifying parent–clinician dissent and evaluating the perceived helpfulness of hospital ethics committees according to the Swiss National NICU Survey Model, which currently uses the Euro-Neonatal Viability Assessment (adapted globally) [14]. Often, perceptions and opinions between neonatal physicians and nurses are different [15].

In a nationwide Lebanese survey, the majority of physicians agreed that parents would like to be informed and to participate in the resuscitation decision [13]. They considered infants at a gestational age of ≤25 weeks not viable, with this opinion being influenced by the age of the physician, years of practice, and hospital level [13]. Similarly, the participants in our study had different opinions related to the limit of viability threshold, which changed after the training course, with years of practice being the most important factor that might influence their decisions regarding the management of such newborns.

According to Zeballos-Sarrato, mortality in the delivery room and late fetal mortality affect the survival rates of preterm infants significantly [16]. However, physician preferences are based on their prognostic abilities [17]. In this context, prognosis is based on “how the infant appears” in the delivery room, considering this to be of predictive value regarding subsequent death or neurological morbidity [17]. According to Singh, only 4% would provide full resuscitation for infants with birth weights of <500 g and a gestational age of 23 weeks [17]. In our study, the perceptions of participants regarding “how the infant appears” in the delivery room changed after the course, with this factor no longer being considered a reliable prognostic tool.

Another issue is that early neonatal deaths are not reported in some countries [18]. For countries where vital registration (VR) is successfully implemented, such data can be used as reported. According to Oza, 57 countries had high-quality VR, compared with 129 countries with low rates of VR. Oza also noted that many lives can be saved with feasible interventions at a low cost, which may improve neonatal and maternity management [18]. There is often misclassification of livebirths and stillbirths, as well as variations in registration with gestational age [13]. Some countries without care for extremely preterm infants may not classify some of these cases as livebirths [18]. This is the case in our country too.

A major concern is the outcomes following the birth of infants at the limit of viability, as well as the pathologies that they develop that affect their quality of life. Participants’ knowledge regarding the comorbidities of such newborns was high even before the course. In a study by Costeloe, from a total of 4004 births among infants at the threshold of viability, 811 infants were admitted to intensive care, with the overall survival rate being 39% [14]. Persistent bradycardia and hypothermia were independent risk factors for death. Of the survivors, 17% had parenchymal cysts and/or hydrocephalus, while 14% developed retinopathy of prematurity [19].

Maternal and neonatal interventions at the threshold of viability may influence outcomes. Maternal interventions such as cesarean delivery or antenatal corticosteroid administration, as well as transfer to a higher-level unit, may influence infants’ chances of survival. Moreover, neonatal interventions—including neonatal intensive care unit (NICU) admission, assisted ventilation, and surfactant and antibiotic administration—have significant impacts on neonatal outcomes. The counseling of women at risk of periviable birth is mandatory [20,21]. Their treatment in the NICU poses a significant financial and emotional burden. The post-NICU complex care of these children can adversely impact parent and offspring wellbeing [10]. Maternal health is particularly affected by such decisions: a cesarean delivery at extremely early gestation is of particularly high risk for the mother [20,21,22,23,24], increasing the possibility of uterine rupture in a subsequent pregnancy [10,25].

We identified differences among physicians based on their perceptions regarding the limit of viability, with variations in reported outcomes and prognostic assessments to the same extent as reported in the literature [3]. However, according to Arzuogo, overall, 68% of healthcare professionals consider that infants at >23 weeks of gestation should be resuscitated, and parents should be involved in the decision-making process. Moreover, 25 weeks of gestation was considered by 51% of responders to be the age at which resuscitation is obligatory, even if the parents refuse. Nevertheless, parental preferences significantly affect the decision. Regional practice variations also exist [1]. In our study, parental involvement in decision-making was not considered an option before the training course, but this changed significantly after the course, although the final decision should be made by the clinician. In the UK, active treatment is recommended from 22 + 0 weeks, after risk assessment and consideration of parental wishes. A multidisciplinary team should be involved in the decision-making, alongside obstetric and neonatal staff and parents [2]. In our research, a multidisciplinary team was considered ideal before the training, while, after training, the respondents reconsidered their position.

The EURONIC project aimed to investigate physicians’ self-reported practices in seven European countries. The ethical issue of end-of-life decisions for newborn infants at high risk of death or severe disability was highly debated [13]. In particular, 61–96% of physicians reported having been involved in setting limits on intensive care due to incurable conditions; the continuation of current treatment was widespread, while the withdrawal of treatment was reported at variable proportions (28–90%). Meanwhile, in France and the Netherlands, healthcare professionals reported the administration of drugs with the aim of ending the patient’s life. Various factors, such as the age of the physician, length of professional experience, and religion, affected the likelihood of reporting non-treatment decisions [26]. In our country, the administration of treatment with the specific purpose of ending life is not an option.

Little empirical evidence is available regarding the factors that affect end-of-life decisions for infants with adverse prognoses in different countries and cultures [27]. Rebagliato explored the variability in neonatal physicians’ attitudes among 10 European countries, covering a total of 1391 physicians. Preserving life at any cost was reported in countries such as Hungary, Estonia, Lithuania, and Italy, while, in the United Kingdom, the Netherlands, and Sweden, the idea that quality of life must be taken into account was more frequently reported by physicians. Other factors that influenced the decision were being female, having had no children, religious background, and working in a NICU. In conclusion, it was found that setting limits on intensive neonatal interventions in cases of a poor neurological prognosis was based on physicians’ attitudes, and the country remained the most important predictor [27].

Infant health severity and family outcomes were also investigated by Grunberg in a review of seventy studies. It is also extremely important to identify families at risk for negative psychosocial sequelae. This depends on the burden of care, parental characteristics, and access to resources [28]. Shared decision-making regarding intensive care for extremely preterm infants between healthcare professionals and parents is the appropriate management approach today [15]. In our study, 79% of physicians and 70% of nurses reported the following difficulties after completing the survey on decision-making: legal constraints, insufficient time for parental counselling, a lack of proper department guidelines, and a lack of solidarity in society. Several differences were reported within the neonatal team, which should be acknowledged in order to avoid potential conflicts [15]. Our participants acknowledged the importance of a multidisciplinary team in such cases, including obstetricians, neonatologists, and midwives, after completing the training course.

The existing guidelines were reviewed by Pignotti, and it was found that a variety of complex aspects give rise to different approaches. However, there is agreement regarding the current recommendations: treatment is considered justifiable at ≥25 weeks, with an individual approach at 23 to 24 weeks, depending on the clinical condition; parents’ wishes are considered; and compassionate care is applicable at ≤22 weeks [29]. The importance of ethical considerations was acknowledged by the healthcare professionals in the current study after the training course.

Variations have been observed among maternal–fetal medicine (MFM) providers regarding with the perceived gestational age for the threshold of viability [30]. The reported threshold of viability varied among survey respondents: the majority reported 24 weeks, namely 55.3%. No significant differences were observed with respect to practitioner age, years in practice, or the hospital level. Significant differences in the reported threshold of viability were noted with respect to practitioner gender, with males tending to have a lower gestational age threshold compared to females (*p* = 0.005) Variations were also noted among practitioners from academic vs. community/private practice settings (*p* = 0.008) [4]. In our survey, no differences were observed between public and private practice.

Written information regarding the mortality and morbidity of infants at the limit of viability given to healthcare professionals encourages them to overestimate the risk. Parental perceptions during counselling are affected by the data received, which subtly shape the decisions that follow [30]. Shared decision-making is a complex process centered on ensuring the best outcome for the newborn, in which clinicians and families face prognostic uncertainty [31].

In Israel, neonatologists posited that resuscitation at a gestational age of 22–24 weeks is not in the best interests of infants, which is in disagreement with the national clinical guidelines. The country’s future guidelines should reflect neonatologists’ opinions [32]. This study also aimed to raise awareness regarding the variability in practitioners’ views on this issue and the importance of constant training.

Communication with parents may have an impact on their decision-making [33]; meanwhile, controversial legal policies can permit physicians to disregard parental preferences when faced with the birth of an extremely premature infant [34]. The Dutch guidelines, for example, lack recommendations regarding decision-making for prenatal counselling [35]. In a survey conducted by Greutzen, the responders preferred joint counselling by both a neonatologist and obstetrician, and decisions on the initiation of care should be made together with the parents [35]. Healthcare professionals can experience conflicts with parental wishes in the face of uncertain outcomes for infants at the limit of viability. The main question remains how to achieve a consensus regarding treatment decisions when the outcome is uncertain and there are divergent views about the best management. However, there must be willingness to work with parents to ensure the best interests of the infant [36]. Most professionals desire standardized guidelines regarding upper (85%) and lower (87%) gestational age limits for offering treatment through fair and transparent decision-making [37].

In a review conducted by Lemyre on the current guidelines, he reported that no published guidelines met the criteria to be considered of high quality [38]. He identified the following key medical factors for decision-making: neurodevelopmental disability, long-term morbidity, quality of life of the child and parents, and maternal mortality. Parental values and preferences should be incorporated into the process. A pre-implementation assessment is the ideal means to identify barriers to implementing new guidelines [38,39]. Multiple factors are involved in infants’ survival and long-term neurodevelopment—for example, gender, birthweight, multiple versus singleton birth, corticosteroid use, and maternal health conditions. There are remarkable differences between countries and even within similar resource environments, while different cultures and local practices generate different survival rates. Instead of applying rigid rules, opportunities for authentic conversation should be sought [8,10,40,41,42]. In our study, we observed inconsistency in practitioners’ approaches, and appropriate training should align their management with international guidelines.

Decisions regarding the management of periviable infants should be made before birth and not be conditional upon the infant’s appearance at birth. In the event of an emergent premature delivery, when little time is available for a dialog, most guidelines recommend resuscitation unless the neonate is below 22 weeks of gestation or severely compromised [7,9,10]. It is recommended not to use disability statistics in parent counselling, as it has been reported that parents’ decisions are not influenced by quoted survival rates or statistics [43]. Moreover, disagreement between obstetricians and neonatologists leads to a 2.4-fold increase in mortality in the first 24 h of life among extremely preterm infants [44]. These differences between obstetricians and neonatologists are common in various countries. Compared to obstetricians, neonatologists are more likely to be interventional in the periviability interval in Finland, Norway, and the UK [10,45], while the opposite is reported in the Netherlands [8,38]. In the Netherlands, national uniformity and the standardization of care limits interventions below 24 weeks of gestation and requires parental consent for active intervention at 24 weeks [10,46], and a personalized approach in parental counselling is recommended [47,48]. Many guidelines refer to the Eunice workshop, which sets the limit of viability at 23 weeks of gestation [49]. In our research, obstetricians and neonatologists did not exhibit significant differences.

The Society for Maternal–Fetal Medicine recommendations for the management of previable and periviable preterm infants should be noted. “Periviable” represents the period at which the fetus may survive outside the uterus with life-sustaining treatment, but still with a high risk of death or severe morbidities [50]. The need for a periviability guideline is clear, as it would provide valuable decision-making support [51]. However, this remains challenging as it depends on the economic development of each country and on the resources allocated to the care of premature neonates [52]. Moreover, it is necessary to distinguish between the limit of neonatal viability—in terms of definition or reporting—and the limit of viability regarding therapeutic decisions.

Our study has some limitations; the most important lies in the small sample size regarding those who agreed to participate to our survey and course. Moreover, dropout bias should be noted (eight participants responded to the first questionnaire, without completing the course), which may reduce the statistical power, but those who dropped out shared the same characteristics as those who remained in the study.

The strength lies in the novelty of our approach: our research included a training course, representing, to our knowledge, the first such research. Moreover, several practitioners from different hospitals participated in our study, and the survey included a wide range of questions.

## 5. Conclusions

The “limit of viability” should be determined by the gestational age, clinical feasibility, prognosis, ethical judgement, and parental values. Future efforts should be made to develop ethically grounded strategies to guide resuscitation decisions in which gestational age is an insufficient parameter. A collaborative, multidisciplinary process is mandatory to ensure that decisions are made in the newborn’s best interests.

While it is impossible to achieve a global consensus in terms of practical strategies, an international dialog should be encouraged. Physicians should adjust their expectations and clinical practice to the local standards. The value of continuing medical education should also be acknowledged.

## Figures and Tables

**Table 1:** Course on dilemmas at the limit of viability in newborns.

Workshop/Type of Questionnaire		Data Captured
1. Demographics	Multiple Choice/Numerical	Profession, NICU level (Level II vs. III/IV), years in practice.
2. Clinical Thresholds and Prognostic Modifiers	Ordinal (22 + 0 to 25 + 6 weeks)	Absolute lowest gestational age for mandatory vs. optional resuscitation. Impact of birth weight, neurodevelopment, or other prognostic factors on the decision.
4. Ethical Dilemmas	Categorical Matrix	Views on withholding vs. withdrawing care; quality of life perceptions.
5. Counseling Style	Multiple Choice	Shared decision-making vs. clinician-led vs. parental choice.

**Table 2:** Demographic and professional characteristics of study participants (*n* = 30).

Age, years	30–40	19 (63.3)
	40–50	5 (16.7)
	50–60	4 (13.3)
	>60	2 (6.7)
Sex	Female	13 (43.3)
	Male	17 (56.7)
Marital status	Married	20 (66.7)
	Single	6 (20.0)
	Divorced	2 (6.7)
	Partnership	2 (6.7)
Religious affiliation	Religious	21 (70.0)
	Not religious	9 (30.0)
Years of practice	<10	6 (20.0)
	11–20 *	14 (46.7)
	21–30 *	7 (23.3)
	31–40 *	3 (10.0)
Type of hospital/practice	Public	26 (86.7)
	Private	4 (13.3)
Annual number of births	400	8 (26.7)
	500	3 (10.0)
	600	5 (16.7)
	700	1 (3.3)
	2000	13 (43.3)
Hospital level	I	1 (3.3)
	II	16 (53.3)
	III	13 (43.3)
Profession	Obstetrician	21 (70.0)
	Neonatologist	9 (30.0)

* Years of practice.

**Table 3:** Changes in participants’ responses regarding the gestational age threshold before and after the educational intervention.

Question/Response	Before, *n* (%)	After, *n* (%)	Stuart–Maxwell *p*	Holm-Adjusted *p*
Q1. Limit of viability threshold			0.0011	0.0068
22 weeks	4 (13.3)	0		
23 weeks	1 (3.3)	0		
24 weeks	14 (46.7)	30 (100)		
28 weeks	11 (36.7)	0		
Q2. WHO lower limit of viability			0.0009	0.0064
22 weeks	19 (63.3)	30 (100)		
24 weeks	11 (36.7)	0		
Q3. Gestational age for treatment/full care			0.0006	0.0044
22 weeks	3 (10.0)	0		
24 weeks	15 (50.0)	30 (100)		
28 weeks	12 (40.0)	0		

**Table 4:** Paired changes in binary questionnaire responses before and after the educational intervention.

Questionnaire Item	Before, Yes *n* (%)	After, Yes *n* (%)	Absolute Paired Change, Percentage Points (95% CI)	Exact McNemar *p* Value	Holm-Adjusted *p* Value
Q7. Do you consider parents’ involvement in treatment decisions?	18 (60.0)	27 (90.0)	+30.0 (+10.0 to +50.0)	0.0117	0.0469
Q8. Would you offer perinatal counselling in cases of pregnancy at risk?	6 (20.0)	30 (100.0)	+80.0 (+63.3 to +93.3)	<0.0001	<0.0001
Q9. Do you think ethical considerations are important in the current guidelines and recommendations?	5 (16.7)	28 (93.3)	+76.7 (+60.0 to +90.0)	<0.0001	<0.0001
Q11. Are you aware of national guidelines and recommendations regarding limits of viability?	11 (36.7)	30 (100.0)	+63.3 (+46.7 to +80.0)	<0.0001	<0.0001
Q15. Should the high economic costs of pre-term infant care be considered in treatment decisions or withdrawal of intensive care?	10 (33.3)	4 (13.3)	−20.0 (−36.7 to −6.7)	0.0313	0.0625
Q16. Do you think that neonatologist stress in neonatal intensive care should be taken into consideration in treatment decisions or withdrawal of intensive care?	5 (16.7)	1 (3.3)	−13.3 (−26.7 to −3.3)	0.1250	0.1250
Q17. Is “how the infant looked” in the delivery room an appropriate tool for evaluating infant outcomes?	10 (33.3)	2 (6.7)	−26.7 (−43.3 to −13.3)	0.0078	0.0391

Note: Data are presented as *n* (%). Pre- and post-course responses were paired at the individual participant level. Within-participant changes were evaluated using the exact McNemar test. Absolute changes are expressed in percentage points with 95% confidence intervals. To account for multiple testing, *p* values were adjusted using the Holm procedure. A two-sided Holm-adjusted *p* value < 0.05 was considered statistically significant.

**Table 5:** Paired changes in multi-categorical decision-making responses after the educational intervention.

Questionnaire Item	Before Distribution, *n* (%)	After Distribution, *n* (%)	Participants with Change in Response Category, *n* (%) (95% CI)	Stuart–Maxwell χ^2^ (df)	*p* Value	Holm-Adjusted *p* Value
Q10. What would you consider more important in the decision: (a) infant experiencing pain or (b) long-term/chronic distress?	a: 5 (16.7); b: 20 (66.7); a + b: 5 (16.7)	b: 1 (3.3); a + b: 29 (96.7)	25/30 (83.3%) (65.3–94.4)	24.23 (2)	<0.0001	<0.0001
Q12. Who should have the final word in the therapeutic decision: clinician, parent, or both?	Clinician: 22 (73.3); Parent: 6 (20.0); Both: 2 (6.7)	Clinician: 30 (100.0)	8/30 (26.7%) (12.3–45.9)	8.00 (2)	0.0183	0.0549
Q13. Who should be trained regarding the infant limit of viability?	O: 5 (16.7); O + N: 18 (60.0); O + N + M: 7 (23.3)	O + N: 3 (10.0); O + N + M: 27 (90.0)	20/30 (66.7%) (47.2–82.7)	20.00 (2)	<0.0001	0.00045
Q14. Which factors influence decision-making in such cases?	a + b + c + d: 2 (6.7); a + b + c + d + e: 3 (10.0); b + c: 9 (30.0); b + c + d: 15 (50.0); c + d: 1 (3.3)	a + b + c + d: 19 (63.3); a + b + c + d + e: 5 (16.7); b + c + d: 6 (20.0)	24/30 (80.0%) (61.4–92.3)	21.93 (4)	0.00021	0.00186

Abbreviations: O = obstetricians; N = neonatologists; M = midwives. For Q14: a = cultural differences; b = religious beliefs; c = education; d = source of information for parents; e = other factors. Note: Data are presented as *n* (%). Pre- and post-course responses were paired at the individual participant level. Changes in multi-categorical response distributions were assessed using the Stuart–Maxwell test of marginal homogeneity. The magnitude of within-participant change is additionally reported as the number and proportion of participants whose response category changed, with 95% confidence intervals. To account for multiple testing across questionnaire outcomes, *p* values were adjusted using the Holm procedure. A two-sided Holm-adjusted *p* value < 0.05 was considered statistically significant.

**Table 6:** Participants’ responses regarding comorbidities in neonates at the limit of viability before and after instructions.

**Before Instructions**					
		Frequency	Percentage	Valid Percentage	Cumulative Percentage
Valid	0	1	3.3	3.3	3.3
	Chronic lung disease, brain injuries, and vision issues	1	3.3	3.3	6.7
	Chronic lung disease, severe brain injuries	11	36.7	36.7	43.3
	Chronic lung disease, severe brain injuries, and vision issues	5	16.7	16.7	60.0
	Chronic lung disease, severe brain injuries, intestinal inflammation, and vision issues	12	40.0	40.0	100.0
	Total	30	100.0	100.0	
**After Instructions**					
		Frequency	Percentage	Valid Percentage	Cumulative Percentage
Valid	Respiratory, neurological, gastrointestinal, cardiovascular, and visual issues	30	100.0	100.0	100.0

**Table 7:** Participants’ responses to the following question: When would you suggest withdrawal of life-sustaining therapies?

**Before Instructions**					
		Frequency	Percentage	Valid Percentage	Cumulative Percentage
Valid	Hopeless prognosis	19	63.3	63.3	63.3
	Hopeless prognosis and extremely severe comorbidities	11	36.7	36.7	100.0
	Total	30	100.0	100.0	
**After Instructions**					
		Frequency	Percentage	Valid Percentage	Cumulative Percentage
Valid	Incompatible with life	12	40.0	40.0	40.0
	Incompatible with life and profoundly poor quality of life	1	3.3	3.3	43.3
	Incompatible with life or profoundly poor quality of life	17	56.7	56.7	100.0
	Total	30	100.0	100.0	

## Data Availability

The original contributions presented in this study are included in the article/Supplementary Materials. Further inquiries can be directed to the corresponding author.

## Supplementary Materials

The following supporting information can be downloaded at https://www.mdpi.com/article/10.3390/children13081111/s1. Questionnaires, informed consent.

## Abbreviations

The following abbreviations are used in this manuscript:

WHO	World Health Organization
ACOG	American College of Obstetricians and Gynecologists
APP	American Academy of Pediatrics
MFM	Maternal–fetal medicine
VR	Vital registration
NICU	Neonatal intensive care unit
