# Supplementary material for: Decision-Making at the Limit of Viability in Newborns: Insights from a Survey of Medical Professionals
Source: Children (Basel). 2026 Aug 19;13(8):1111. doi: 10.3390/children13081111 (PMC13511257; doi:10.3390/children13081111)
Supplement: Supplementary file 1 [file children-13-01111-s001.zip › children-4483434-supplementary.pdf]

Improving Survival at the Threshold of Viability: Insights from a Survey of Medical Professionals and the Role of Continuing Medical Education

Questionnaire limits of viability

1. What is the limits of viability threshold?
2. Where sets the World Health Organization lower limit of viability?
3. At what gestational age would you prefer treatment/full care?

4. Participants characteristics:

a-age 30-40,4—50,50-60,>60

b-gender: male, female, I prefer not to declare

c-marital status: married, in a partnership, divorced, single

d-religion: religious, not religious

e-work experience: no of years

f-type of healthcare experience: public hospital, private practice

g-hospital volume of birth

h-hospital level: I, II, III

i-are you obstetrician or neonatologist?

5.What are the main morbidities in periviable infants?

6.When would you suggest withdrawal of life-sustaining therapies?

7.Do you consider parents involvement in treatment decisions?

8.Would you offer perinatal counselling in case of pregnancy at risk?

9.Do you think ethical considerations important in current guidelines and recommendations?

10.What would you consider more important in decision:

a-infants experiencing pain or

b- long-term or chronic distress?

11.Are you aware of national guidelines and recommendation regarding limits of viability?

12.Who should have the final word in the therapeutic decision: the clinician or the parent?

13.Who do you think should be trained regarding infant s limit of viability, obstetricians, neonatologists, midwives?

14.What do you think influences the decision in threating such cases:

a.-cultural differences,

b-religious beliefs,

c-education,

d-source of information for parents,

e-other?

15.Do you think that high economic costs in preterm infants care should be taken into consideration in treatment decision/ or withdrawing intensive care?

16.Do you think that neonatologist s stress in neonatal intensive care should be taken into consideration in treatment decision/ or withdrawing intensive care?

17.Do you think that "how the infant looked" in the delivery room is a proper tool in the evaluation of infants outcome?
